# Regulation of striatal dopamine responsiveness by Notch/RBP-J signaling

**DOI:** 10.1038/tp.2017.21

**Published:** 2017-03-07

**Authors:** M Toritsuka, S Kimoto, K Muraki, M Kitagawa, T Kishimoto, A Sawa, K Tanigaki

**Affiliations:** 1 Research Institute, Shiga Medical Center, Shiga, Japan; 2 Department of Psychiatry, Nara Medical University, Faculty of Medicine, Nara, Japan; 3 Department of Molecular and Tumor Pathology, Chiba University, Graduate School of Medicine, Chiba, Japan; 4 Department of Psychiatry and Behavioral Sciences, Johns Hopkins University, School of Medicine, Baltimore, MD, USA

## Abstract

Dopamine signaling is essential for reward learning and fear-related learning, and thought to be involved in neuropsychiatric diseases. However, the molecular mechanisms underlying the regulation of dopamine responsiveness is unclear. Here we show the critical roles of Notch/RBP-J signaling in the regulation of dopamine responsiveness in the striatum. Notch/RBP-J signaling regulates various neural cell fate specification, and neuronal functions in the adult central nervous system. Conditional deletion of RBP-J specifically in neuronal cells causes enhanced response to apomorphine, a non-selective dopamine agonist, and SKF38393, a D1 agonist, and impaired dopamine-dependent instrumental avoidance learning, which is corrected by SCH23390, a D1 antagonist. RBP-J deficiency drastically reduced dopamine release in the striatum and caused a subtle decrease in the number of dopaminergic neurons. Lentivirus-mediated gene transfer experiments showed that RBP-J deficiency in the striatum was sufficient for these deficits. These findings demonstrated that Notch/RBP-J signaling regulates dopamine responsiveness in the striatum, which may explain the mechanism whereby Notch/RBP-J signaling affects an individual's susceptibility to neuropsychiatric disease.

## Introduction

Dopamine (DA) signaling is highly related to reward-related learning, which also contributes to fear-related learning.^1, 2^ Abnormalities in DA neurotransmission are thought to exist in neuropsychiatric diseases. In spite of recent progress, much remains to be elucidated concerning the molecular mechanisms underlying the regulation of DA responsiveness.

Notch4 has been reported to be associated with schizophrenia.^3, 4, 5^ However, it remains unknown whether deficits in Notch/RBP-J signaling are involved in schizophrenia-like behavioral abnormalities or not. Notch/RBP-J signaling is highly conserved and known to play pivotal roles in various aspects of developmental neural cell fate specification,^6, 7, 8, 9, 10^ dendrite morphogenesis^11, 12, 13^ and neuronal functions in the adult central nervous system.^14, 15, 16, 17, 18^ Notch/RBP-J signaling regulates synaptic plasticity and memory formation in both invertebrates and vertebrates.^14, 15, 16, 17, 18, 19^ In the absence of Notch activation, RBP-J functions as a transcriptional repressor.^9, 20^ Interaction of the Notch receptor with Delta-Serrate ligand family activates Notch signaling and leads to proteolytic processing of Notch by γ-secretase, which consists of Presenilin, Niscartin, Pen2 and Aph1.^21, 22^ The released intracellular domain of Notch translocates to the nucleus and upregulates the transcription of its target genes by interacting with RBP-J and a co-activator, MamL1.^23, 24, 25, 26^ It has been reported that reduced expression of Aph1b by genomic recombination caused hyper-responsiveness to apomorphine in rats.^27^ Aph1b/c knockout mice also show hypersensitivity to amphetamine.^28^ However, the diverse substrates of γ-secretase made it difficult to delineate the molecular mechanisms underlying these behavioral abnormalities.

Here we believe we provide the first evidence that Notch/RBP-J signaling is essential for the regulation of DA responsiveness. Neuron-specific loss of Notch/RBP-J signaling leads to a deficit in DA-dependent instrumental avoidance learning and hyper-responsiveness to apomorphine and SKF38393, a D1 agonist. Neuron-specific deletion of RBP-J caused reduction in DA release in the striatum, and the number of dopaminergic neurons in the substantia nigra compacta (SN) and ventral tegmental area (VTA) decreased. Furthermore, lentivirus-mediated gene transfer experiments showed critical roles of RBP-J in the striatum to regulate the responsiveness to DA.

## Materials and methods

### Animals

*RBP-J-floxed* mice,^29^ *CamkII-Cre* (line 159) transgenic mice^30^ and *ROSA26R-lacZ* Cre reporter^31^ mouse lines were previously described in detail. Mice were maintained on the C57BL/6N genetic background for at least 10 generations. Male, 3–5-month-old mice were used for behavioral analysis. Mouse colonies were maintained in accordance with the protocols approved by the Committee on Animal Research at Research Institute, Shiga Medical Center.

### Immunohistochemistry of tissue sections

Under deep pentobarbital anesthesia, mice were perfused with phosphate-buffered saline (PBS), followed by 4% paraformaldehyde and 0.1% glutaraldehyde in PBS. Brains were removed and fixed for 12 h in the same fixative at 4 °C and then soaked in 30% sucrose in 0.1 m phosphate buffer for 12 h at 4 °C. The brains were frozen in tissue-tek OCT compound (Sakura Finetechnical, Tokyo, Japan) and cut at 40 μm thickness. Cryostat free-floating sections were incubated with primary antibodies for 24 h at 4 °C after blocking for 30 min at room temperature with 5% donkey serum (Millipore, Billerica, MA, USA). The sections were incubated for 1 h at room temperature with secondary antibodies with 5% donkey serum (1:1000, Molecular Probes, Eugene, OR, USA). The primary antibodies used were anti-TH (1:300, MAB318, Millipore), anti-NeuN (1:200, MAB377, Millipore), anti-S100β (1:1800, S2532, Sigma, St Louis, MO, USA), anti-MBP (1:180, AB980, Millipore) and anti-DARPP32 (1:900, AB1656, Millipore) antibodies. Slides were examined with an Olympus confocal laser scanning microscope (FV-300, Olympus, Tokyo, Japan).

### LacZ staining

Under deep pentobarbital anesthesia, mice were perfused with PBS, followed by 2.2% formaldehyde and 0.2% glutaraldehyde in PBS. Brains were removed and fixed for 1 h in the same fixative at 4 °C and then soaked in 30% sucrose in 0.1 m phosphate buffer for 12 h at 4 °C. The brains were frozen in tissue-tek OCT compound and cut at 30 μm thickness. Sections were subjected to lacZ staining overnight at 37 °C. LacZ staining solution contains 0.5 mg ml^−1^ Bluo Gal (Invitrogen, Carlsbad, CA, USA), 3 mm K_4_Fe(CN)_6_, 3 mm K_3_Fe(CN)_6_ and 1 mm MgCl_2_ in PBS.

### Cell counting

Quantification of TH^+^ neurons was performed on 3,3-diaminobenzidine (DAB)-stained 40 μm serial sections spanning the SN and the VTA (Vectastain ABC Kit Elite, Vector Laboratories, Burlingame, CA, USA). Sections were obtained in semi-series, using two in every three sections. A Zeiss Axiovert 200M microscope and AxioCam MRm (Carl Zeiss, Jena, Germany) were used to count TH^+^ cells. Cell counts from serial sections were corrected and extrapolated for whole volumes of the SN and VTA by Cavelieri's method (reference). To avoid a repeat cell count, cells at the bottom of a section were accurately compared with those of the top of the next section.

### Active avoidance

For this analysis, we used a two-way shuttle box (Med Associates, St. Albans, VT, USA). Mice were given one training session each day for 4 consecutive days. Each session consisted of an adaptation period of 5 min followed by 50 trials separated by an intertrial interval of 20±8 s. In each trial, a conditioned stimulus (CS) of a light and a tone (2900 Hz, 65 dB) preceded the onset of an unconditioned stimulus (US) of a 0.5 mA electrical shock. The US continued until the mouse escaped to the other compartment. If the mouse did not move to the other compartment, the US lasted 10 s along with the CS. If the mouse moved within 10 s after the CS, the CS was stopped and no US was given. An avoidance response was defined when the mouse moved to the opposite compartment after the CS started but before the US was delivered. The number of avoidances was used to measure the learning performance. The number of crossings between chambers during intertrial intervals was measured to assess the spontaneous activity of the mouse. For acute behavioral studies, 100 mg kg^−1^ *N*-*S*-phenyl-glycine-t-butyl ester (DAPT; Tocris, Bristol, UK), 0.01 mg kg^−1^ SCH23390 (Sigma) or 0.05 mg kg^−1^ haloperidol (Sigma) was administrated 3 h, 10 min or 30 min before experiments, respectively.

### Passive avoidance

For this analysis, we used a passive avoidance chamber (Med Associates). The mouse was placed in a box consisting of two different compartments separated by a shutter, that is, illuminated and dark compartments. On the first day, the mouse was kept in the light compartment. Ten seconds later, the door to the dark compartment was opened. When the mouse crossed to the dark compartment, the automatic door was closed and the mouse received an electrical shock (0.5 mA, 5 s). Twenty-four hours later, each mouse was placed again in the light compartment and the latency to enter the dark compartment was recorded.

### Open-field test

Locomotor activity was measured using open-field apparatus (40 × 40 × 40 cm) equipped with photocells (beam spacing 2.5 cm). Automatic recording of stereotypy count was analyzed using the VersaMax system (Accuscan Instruments, Columbus, OH, USA). Data were collected for 15 min. Stereotypy count is the number of times a mouse broke the same beam.

### Stereotaxic surgery

Injections of the lentiviruses Lenti-CaMKII-Cre vector and Lenti-CAG-GFP vector were performed stereotaxically in 8–10-week-old mice. Mice were anesthetized using 2.5% avertin solution. The coordinates of the injection sites were 0.2 mm anterior and 2.5 mm lateral to the bregma at a depth of 2.5 mm; 0.7 mm anterior and 2.75 mm lateral to the bregma at a depth of 2.5 mm; 0.5 mm posterior and 1.5 mm lateral to the bregma at a depth of 2.0 mm and 2.5 mm; 0.5 mm posterior and 2.25 mm lateral to the bregma at a depth of 2.0 mm and 2.5 mm; 1.2 mm posterior and 1.25 mm lateral to the bregma at a depth of 2.0 mm; and 1.2 mm posterior and 2.0 mm lateral to the bregma at a depth of 2.0 mm. A total of 0.5 μl of purified virus was delivered on each side over a 3-min period. Lentivirus-infected mice were used for experiments at least 4 weeks after surgical operations for recovery. All procedures were performed according to the guidelines on animal experiments of Shiga Medical Center.

### Quantification of striatal catecholamines and their metabolites

Mice were decapitated and the brains were quickly removed. Wet striatal tissues were homogenized in 0.2 m perchloric acid buffer containing 100 μm EDTA 2 Na and 1 μg ml^−1^ isoproterenol (100 μl per 1 mg wet weight tissue) and kept on ice for 30 min at 4 °C. Isoproterenol was added as an internal standard. The supernatants of homogenates were collected after 15 000 r.p.m. centrifugation for 15 min and analyzed using a high-performance liquid chromatography system with an electrochemical detector (HTEC-500, Eicom, Kyoto, Japan) for the analysis of DA, 4-dihydroxyphenylacetic acid (DOPAC), homovanillic acid (HVA), serotonin (5HT) and 5-hydroxyindolacetic acid (5-HIAA). The column used for the separation was a SC-5ODS (Eicom). The mobile phase was 0.1 m sodium phosphate buffer (0.1 m NaH_2_PO_4_:0.1 m Na_2_HPO_4_=1000:160, v/v), 1% methanol, 500 mg l^−1^ sodium sulfonate and 50 mg l^−1^ EDTA.

### Microdialysis and high-performance liquid chromatography analysis

A guide cannula (AG-2, Eicom) with length 4 mm, inner diameter 0.4 mm and outer diameter 0.5 mm was implanted into the striatum (*A*, 0.2 mm; *L*, 2 mm; and *V*, 1.5 mm from the bregma) and fixed to the skull with dental cement. Following surgery, the animals were allowed to recover for 1 day before beginning of the experiment. The microdialysis probe (A-1-4-01, Eicom) with an active dialysis membrane (1 mm long; inner diameter, 0.20 mm; outer diameter, 0.22 mm; cut-off value, 50 kDa) was inserted carefully into the striatum. The implanted microdialysis probe was perfused with Ringer's solution at 1 μl min^−1^. The dialysate collected during the first 2 h was discarded to ensure a stable baseline of DA. Twenty-microliter samples of perfusate were collected at 20 min intervals. DA content in the dialysate was quantified in the same way as for tissue homogenates.

### Quantitative reverse transcription-PCR

Total RNA was extracted from the striatum and the ventral midbrain containing the SN and VTA using RNA easy Mini kit (Qiagen, Hilden, Germany). Complementary DNA was obtained using a PrimeScript 1st strand cDNA synthesis kit (Takara, Shiga, Japan). Gene expressions were quantified using the Roche Universal Probe Library method. Quantitative real-time PCR was performed on a LightCycler 480 system (Roche, Basel, Switzerland). All of the data were analyzed by using GAPDH levels as reference (Universal Probe Library Mouse GAPD Gene Assay, Roche). PCR primers and probes are listed in Supplementary Table1.

### Radioligand binding assays

The striatum was removed and frozen at −80 °C and homogenized in 250 volumes (W/V) buffer containing 50 mm Tris-HCl (pH 7.4), 1 mm EDTA, 5 mm KCl, 1.5 mm CaCl_2_, 4 mm MgCl_2_, 120 mm NaCl (for D1 receptors) or 10 mm NaCl (for D2 receptors) using teflon-glass homogenizer (500 r.p.m., 10 times). Aliquots of the buffer (0.2 ml) with 0–18 mm DA were placed in the test tubes and followed by the addition of 0.1 ml of 4 nm [^3^H] SCH23390 (for D1 receptors, 65.0 Ci mmol^−1^, GE Healthcare, Waukesha, WI, USA) or 8 nm [^3^H] raclopride (for D2 receptors, 62.2 Ci mmol^−1^, PerkinElmer, Waltham, MA, USA) and 0.1 ml of the homogenized tissues and incubated at room temperature for 2 h. The incubates were filtered through buffer-presoaked 96-well-MultiScreen FB filter plates (Millipore) using a MultiScreenHTS-Vacuum Manifold (Millipore). The filters were washed five times with 200 μl of ice-cold assay buffer and completely dried, and 40 μl per well MicroScint (PerkineElmer) was added to the dried filter plates. After 5 h, ^3^H radio activity was measured by a liquid scintillation counter (MLC-2001; Aloka, Tokyo, Japan). The binding maximum capacity (Bmax) was determined by fitting specific binding by non-linear regression using GraphPad Prism 5.01 software (GraphPad Software, La Jolla, CA, USA).

### Statistical analysis

The Shapiro–Wilk test for a normal distribution of variables and the Bartlett test for the equality of variance were performed using JMP 12 (SAS Institute, Cary, NC, USA). Statistical evaluation was performed either by unpaired Student's *t-*test or by repeated two-way analysis of variance (ANOVA; StatView, SAS Institute). All *t*-tests were two-tailed. Because of the exploratory nature of the study, no formal power or sample size estimation was performed. Sample size for the analysis was decided based on published studies. Data are presented as means ±s.d. or s.e.m. Statistical significance was set at *P*<0.05. Randomization was not used because allocation to experimental groups was based on the genotype of the animals. An experimenter was blinded to the animal genotype.

## Results

### Normal anatomical structure of the brains of neuron-specific RBP-J-deficient mice

**Figure 1:**
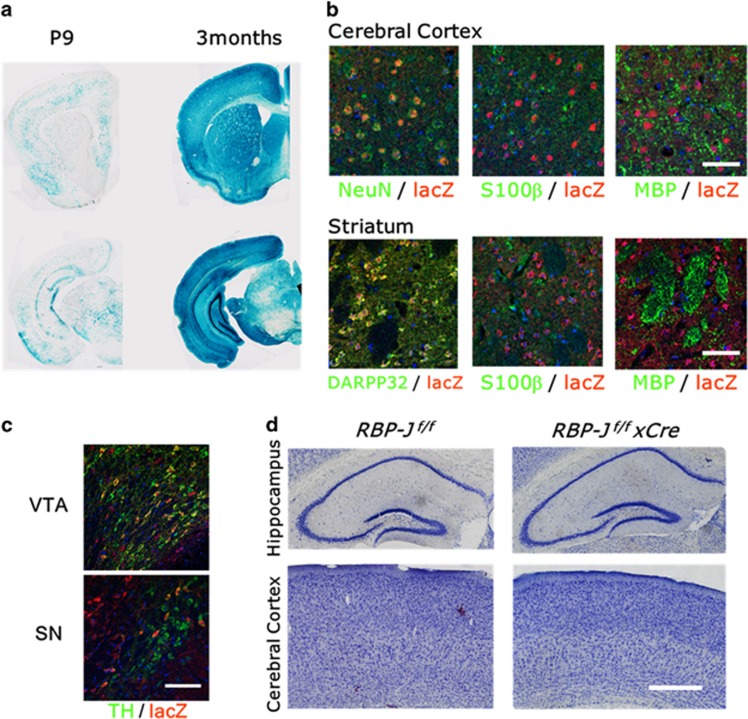
Anatomical analysis of neuron-specific *RBP-J*-deficient mice. (**a**) LacZ staining of coronal sections of *R26R-lacZ* x *CamKII-cre* postnatal day 9 (P9) and 3-month mice. (**b**, **c**) Immunofluorescence analysis for TH (green), NeuN (green), S100b (green), MBP (green), DARPP32 (green) and lacZ (red) in the cerebral cortex and the striatum (**b**), and the ventral tegmental area (VTA) and the substantia nigra (SN) (**c**) of *R26R-lacZ* × *CamKII-cre* mice. Scale bars, 50 μm (**b**); 100 μm (**c**). (**d**) Representative images of Nissle-stained sections of 3-month *RBP-J* ^*f/f*^ and *RBP-J* ^*f/f*^ × *Cre* mice focused on the hippocampus and cerebral cortex. Scale bar, 500 μm.

To examine the neuronal functions of RBP-J in the adult central nervous system, we crossed *RBP-J*-floxed (*RBP-J*^*f/f*^) mice^29, 32^ with the *CamKII-Cre* 159 line (*CamKII-Cre*).^30^ We adopted this transgenic line because its Cre-mediated deletion was observed in the striatum as well as in the cortex and hippocampus (Figure 1a), whereas other *CamKII-Cre* transgenic lines showed extremely low deletion in the striatum.^33, 34^ Immunohistochemical analysis of *R26R-lacZ* Cre reporter mice crossed with these *CaMKII-Cre* mice showed high Cre expression specifically in neuronal cells. Cre-mediated deletion was observed in 93.7±3.3% of DARPP32^+^ medium spiny neurons in the striatum and 70.6±2.4% of NeuN^+^ cortical neurons but not in S100β^+^ astrocytes and MBP^+^ oligodendrocytes (Figure 1b). In contrast, a lower percentage of TH^+^ dopaminergic neurons in the VTA (22.9±6.5%) and the SN compacta (9.3±2.3%) expressed Cre (Figure 1c). Nissle staining of brain sections indicated normal brain structures of the hippocampus and the cerebral cortex of neuron-specific RBP-J knockout mice (Figure 1d).

### Neuron-specific RBP-J-deficient mice have deficits in active avoidance but not in passive avoidance

**Figure 2:**
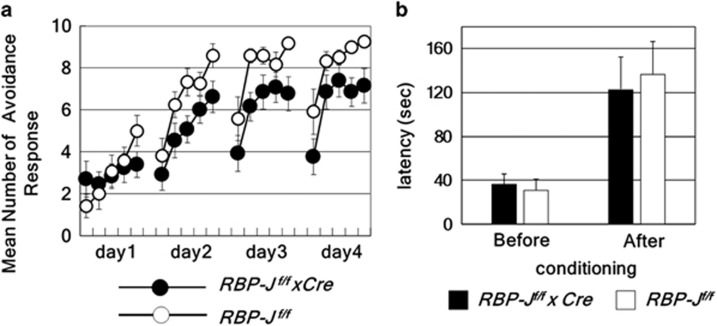
Neuron-specific RBP-J deletion causes deficits in instrumental learning. (**a**) Active avoidance behavioral responses of *RBP-J* ^*f/f*^ × *Cre* mice are indicated. The data are the mean±s.e. of the number of avoidance responses per 10 trials for 12–13 mice. (**b**) Passive avoidance learning of *RBP-J* ^*f/f*^ × *Cre* mice is indicated as step-through latency of 10–12 mice. The data are the mean±s.d. *RBP-J* ^*f/f*^ × *Cre* and *RBP-J* ^*f/f*^ control mice were probed 24 h after training.

To assess the effects of neuron-specific deletion of *RBP-J* in DA-dependent instrumental learning, neuron-specific *RBP-J* -deficient mice and control mice were evaluated using two-way active avoidance tasks, which are a measure of associative learning and reinforcer-driven DA-dependent instrumental learning.^35, 36, 37, 38, 39, 40^ Active avoidance requires not only associating light and tone cues with an electric shock but also learning a strategy to avoid the electric shock. Neuron-specific *RBP-J*-deficient mice showed deficits in learning this task compared with control mice (genotype, F_1,23_=4.65, *n*=12–13 per group, *P*=0.042, repeated two-way ANOVA; Figure 2a). Next, to discriminate whether associative learning or instrumental learning was impaired in neuron-specific *RBP-J*-deficient mice, we performed passive avoidance tasks, wherein control and neuron-specific *RBP-J*-deficient mice showed no difference in step-through latency (*n*=10–12 per group, *P*=0.75, Student's *t*-test) (Figure 2b), which suggests that pain sensitivity and associative learning are intact in neuron-specific *RBP-J*-deficient mice.

### Dysregulation of Notch signaling can cause deficits in active avoidance

**Figure 3:**
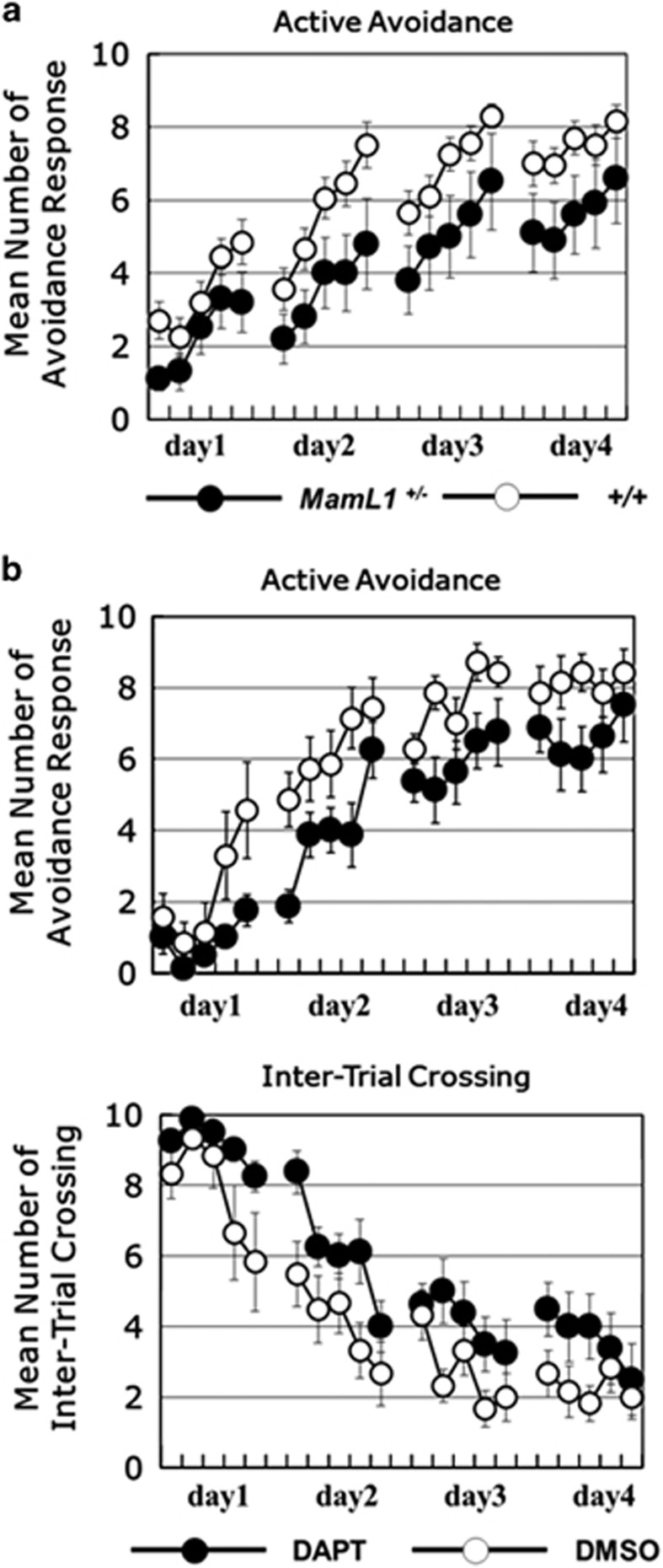
Dysregulation of Notch signaling causes deficits in instrumental learning. (**a**) Active avoidance behavioral responses of *MamL1* KO heterozygous mice are indicated. The data are the mean±s.e.m. of the number of avoidance responses per 10 trials for 10–20 mice. (**b**) The effects of *N*-*S*-phenyl-glycine-t-butyl ester (DAPT), a Notch signaling inhibitor on active avoidance behavioral responses. DAPT (100 mg kg^−1^) was administrated subcutaneously 3 h before behavioral testing. The data are the mean±s.e.m. of the number of avoidance responses and intertrial crossings per 10 trials for 8–10 mice.

RBP-J has been reported to have two functions as a transcriptional repressor and an activator.^9, 20^ To identify which function of RBP-J is indispensable for instrumental learning, we examined the effects of the inhibition of Notch signaling. MamL1 is a co-activator of Notch signaling, which is essential for RBP-J-mediated transcriptional activation.^25, 26^ We first examined the behavioral phenotypes of *MamL1*-knockout heterozygous mice.^41, 42^ *MamL1*-knockout heterozygous mice also showed deficits in active avoidance learning (genotype, F_1,28_=4.81, *n*=10–20 per group, *P*=0.037, repeated two-way ANOVA; Figure 3a). Next, we investigated the effects of a Notch inhibitor, DAPT on instrumental learning. DAPT also significantly attenuated avoidance responses (treatment, F_1,13_=5.13, *n*=7–8 per group, *P*=0.0413, repeated two-way ANOVA; Figure 3c), although the DAPT-treated group showed a trend to enhanced intertrial crossing behavior (Figure 3b), showing that DAPT treatment did not reduce basal activity of the mice. Taken together, these data demonstrated that Notch signaling deficiency also impairs instrumental avoidance learning, suggesting that a transcriptional activation function of RBP-J is responsible for the learning.

### Striatum-specific RBP-J-deficient mice have deficits in active avoidance

**Figure 4:**
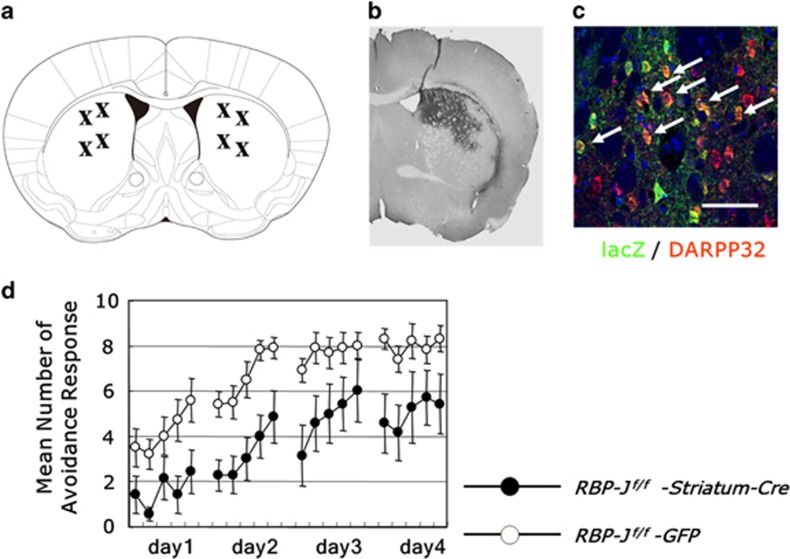
Striatum-specific *RBP-J* deletion causes deficits in instrumental learning. (**a**) Schematic illustration of injection sites in the dorsal striatum, which are modified from the mouse atlas (Paxinos and Franklin, 2001).^65^ (**b**) LacZ staining of coronal sections of *R26R-lacZ* mice infected by Lenti-CamKII-Cre demonstrating spatial selectivity. (**c**) Immunohistochemical studies for lacZ (green) and DARPP32 (red) expression in a representative *R26R-lacZ* mice infected by Lenti-CamKII-Cre. White arrows indicate lacZ-expressing DARPP32^+^ cells. Scale bar, 50 μm. (**d**) Active avoidance behavioral responses of Lenti-CamKII-Cre or Lenti-GFP-infected *RBP-J* ^*f/f*^ mice are indicated. The data are the mean±s.e.m. of the number of avoidance responses per 10 trials for 7–10 mice.

Essential neural substrates of active avoidance are the amygdala, the hippocampus, the infralimbic cortex and the striatum.^43, 44, 45, 46, 47, 48, 49^ Particularly, the dorsal striatum is involved in procedural and instrumental learning.^50, 51^ To examine whether RBP-J in the striatum is necessary for active avoidance learning, we bilaterally injected Cre-expressing lentiviruses with a neuron-specific Calmodulin-dependent kinase II (CaMKII) promoter (Lenti-CaMKII-Cre) to the dorsal striatum of *RBP-J*^*f/f*^ mice, using enhanced green fluorescent protein as a control (Figure 4a). Immunohistochemical analysis of lentivirus-infected *R26R-lacZ* Cre reporter mice confirmed Cre-mediated deletion in the 56.8±5.6% of DARPP32^+^ medium spiny neurons in the infected regions of the dorsal striatum (Figures 4b and c). Striatum-specific *RBP-J* deficiency also caused deficits in active avoidance learning (virus type, F_1,15_=9.03, *n*=7–10 per group, *P*=0.0089, repeated two-way ANOVA; Figure 4d). These results revealed a role of RBP-J for the striatum in striatum-dependent instrumental learning.

### Effects of dopaminergic agonists and antagonists on behavioral abnormalities of neuron-specific RBP-J-deficient mice

**Figure 5:**
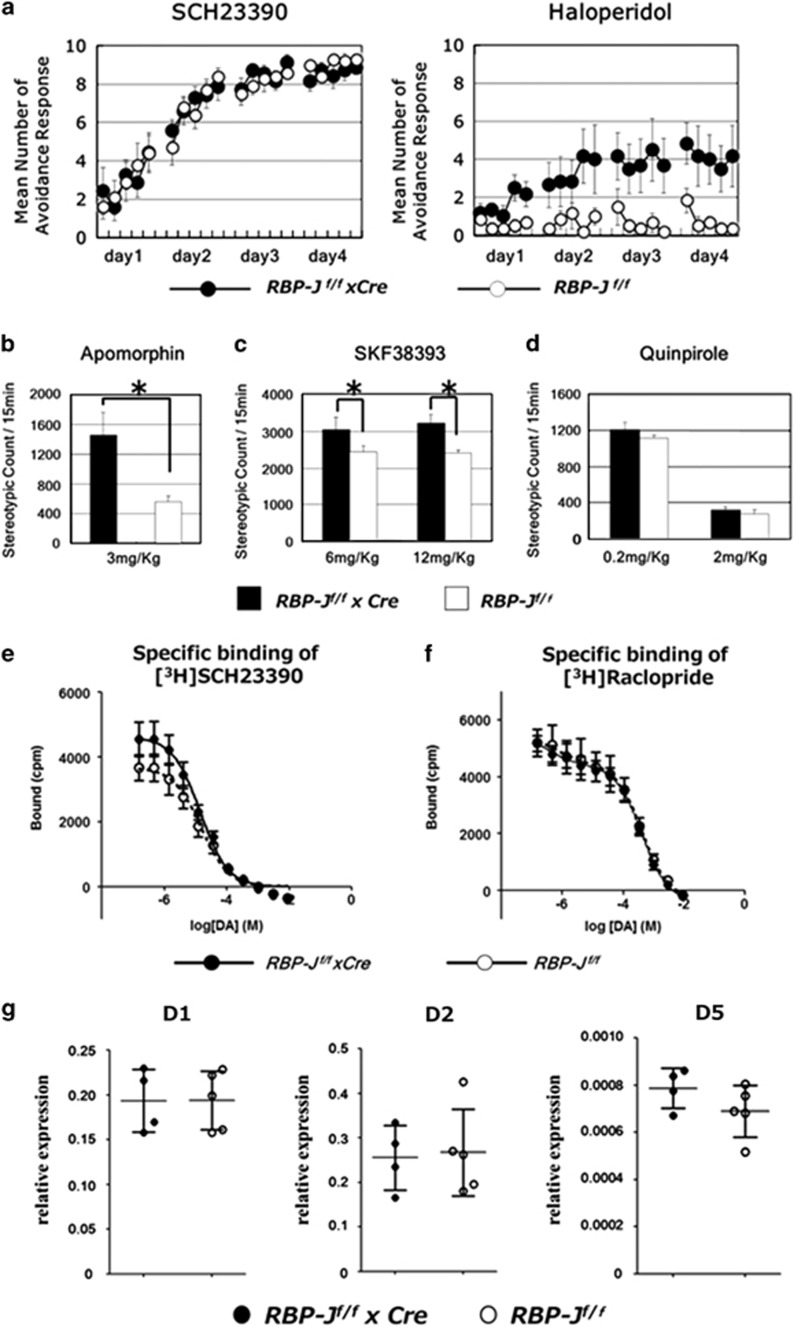
Effects of dopaminergic agonists and antagonists on behaviors of neuron-specific *RBP-J*-deficient mice. SCH23390 but not haloperidol ameliorated the deficits of *RBP-J* ^*f/f*^ × *Cre* mice in the active avoidance learning. (**a**) SCH23390 (0.01 mg kg^−1^) or haloperidol (0.05 mg kg^−1^) was subcutaneously or intraperitoneally injected to male *RBP-J* ^*f/f*^ × *Cre* and control mice. Active avoidance was performed 10 or 30 min after drug administration and the data are the mean±s.e.m. of the number of avoidance responses per 10 trials for six mice. (**b**–**d**) Enhanced response of male *RBP-J* ^*f/f*^ × *Cre* mice to apomorphine (3 mg kg^−1^) (**b**) and SKF38393 (6 and 12 mg kg^−1^) (**c**) but not to quinpirole (0.2 and 2 mg kg^−1^) (**d**). Apomorphine, SKF38393 and quinpirole were subcutaneously injected. Stereotypy count is shown in for 15 min after drug administration. Values are the mean±s.e.m. for 5–8 male mice. *P=0.018, **P=0.047, ***P=0.037. (**e**, **f**) Neither dopamine receptor D1 binding nor D2 binding was affected in the striatum of neuron-specific RBP-J-deficient mice. Dopamine competitions of [^3^H] SCH23390 or [^3^H] raclopride binding are shown. Values are the mean±s.e.m. for three male mice. (**g**) Real-time PCR quantitation of *Dopamine receptor D1, D2 and D5* mRNA levels in *RBP-J* ^*f/f*^ × *Cre* or *RBP-J* ^*f/f*^ mice. Results were normalized to GAPDH abundance. Each point represents a single mouse, with the lines representing the mean±s.d. of each group.

Dopaminergic signaling in the striatum is indispensable for conditioned avoidance.^46, 52^ We first examined the effects of dopaminergic antagonists on the deficits of neuron-specific *RBP-J*-knockout mice in active avoidance learning. A D1 receptor antagonist, SCH23390, restored the learning abnormalities and abolished the difference (genotype, F_1,15_=4.98 × 10^−4^, *n*=7–10 per group, *P*=0.98, repeated two-way ANOVA; Figure 5a). In contrast, haloperidol, a D2 receptor antagonist, inhibited the conditioned avoidance in control mice but did not affect that of neuron-specific *RBP-J*-knockout mice (genotype, F_1,10_=8.46, *n*=6 per group, *P*=0.016, repeated two-way ANOVA; Figure 5a). In addition, in the general locomotion neuron-specific *RBP-J-*knockout mice showed enhanced response to apomorphine, a non-selective DA agonist, and SKF383593, a D1 agonist, but not to quinpirole, a D2 agonist (apomorphine: *n*=6 per group, *P*=0.018, SKF383593 (12 mg): *n*=5–7 per group, *P*=0.037, quinpirole (2 mg): *n*=5–7 per group, *P*=0.12, Student's *t*-test; Figures 5b–d). Taken together, our data suggest that neuron-specific loss of RBP-J caused dopaminergic dysfunction, leading to deficits in DA-dependent instrumental learning.

To gain further insights into the mechanisms of dopaminergic dysfunctions caused by RBP-J deficiency, we examined DA receptors in the striatum of neuron-specific *RBP-J*-knockout mice. The DA/[^3^H] SCH23390 or [^3^H] raclopride competition assay revealed similar D1-like and D2-like receptor binding (Figures 5e and f; Supplementary Figures 1a and b). Quantitative reverse transcription-PCR also showed that the expression levels of D1, D2 and D5 were not affected by the absence of RBP-J (Figure 5g).

**Figure 6:**
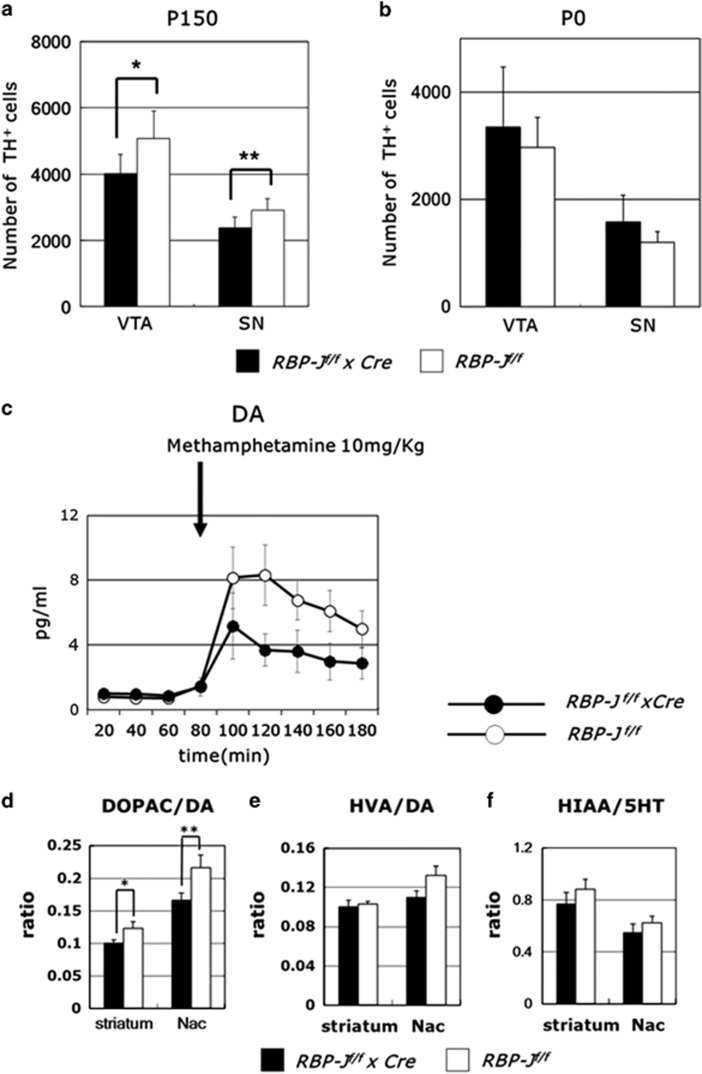
Dopamine release abnormalities in the striatum of neuron-specific *RBP-J-*deficient mice. (**a**, **b**) The decreased number of TH^+^ neurons at P150 (**a**) but not at P0 (**b**) in the VTA and SN of neuron-specific *RBP-J*-deficient mice. The data are the mean±s.e. from four to six mice (**a**) and three to five mice (**b**). *P=0.0433, **P=0.044. (**c**) Effects of methamphetamine on the striatum extracellular dopamine in *RBP-J* ^*f/f*^ × *Cre* mice. Three 20 min baseline fractions were collected before methamphetamine (10 mg kg^−1^, i.p.) injection and thereafter 20 min fractions were collected for 180 min. Data are means±s.e.m. from four to five mice. (**d**–**f**) Decreased dopamine turnover in the striatum and the nucleus accumbens of neuron-specific *RBP-J*-deficient mice. The ratios of the content of dihydroxyphenylacetic acid (DOPAC) to DA (**d**), homovanillic acid (HVA) to DA (**e**) and 5-hydroxyindole acetic acid (5-HIAA) (**f**) to serotonin (5HT) of *RBP-J* ^*f/f*^ × *Cre* mice. The data are the mean±s.e.m. from 8 to 11 mice. *P=0.033, **P=0.032. SN, substantia nigra compacta; VTA, ventral tegmental area.

Next, we measured the number of dopaminergic neurons in the VTA and the SN. Stereological analysis demonstrated that neuron-specific RBP-J deficiency reduced the number of dopaminergic neurons at P150 but not at P0, suggesting an essential roles of RBP-J in the survival of dopaminergic neurons (Figures 6a and b). However, the effects of the reduced dopaminergic neurons seem negligible, because DA contents in the striatum was not affected (*RBP-J* ^*f/f*^ × *Cre* mice: 22.8±2.7 ng mg^−1^, *RBP-J* ^*f/f*^ mice: 24.2±4.8 ng mg^−1^, *n*=8–11 per group, *P*=0.78, Student's *t*-test). It has been reported that Sonic hedgehog released from dopaminergic neurons in the midbrain regulate the expression of glial cell line-derived neurotrophic factor in the striatum, which is indispensable for dopaminergic neuron survival.^53^ However, Sonic hedgehog–glial cell line-derived neurotrophic factor pathway was not affected in the absence of RBP-J (Supplementary Figure 2).

### Abnormalities in DA metabolites in neuron-specific RBP-J-deficient mice

To examine the functions of dopaminergic neurons, we performed *in vivo* microdialysis in neuron-specific RBP-J-deficient mice. In neuron-specific RBP-J-deficient mice, methamphetamine-induced DA release in the striatum was drastically reduced (genotype × time interaction, F_8,56_=2.27, *n*=4–5 per group, *P*=0.036, repeated two-way ANOVA; Figure 6c). Next, we examined the effects of neuron-specific loss of RBP-J on DA and 5HT metabolism in the striatum and the nucleus accumbens of neuron-specific *RBP-J*-deficient mice. The ratio of DOPAC to DA significantly decreased in neuron-specific *RBP-J*-deficient mice, but neither HVA/DA nor hydroxyindole acetic acid (HIAA)/5HT was changed (*n*=8–11 per group, striatum: DOPAC/DA: *P*=0.033, HVA/DA: *P*=0.73, HIAA/5HT: *P*=0.36, nucleus accumbens: DOPAC/DA: *P*=0.032, HVA/DA: *P*=0.065, HIAA/5HT: *P*=0.41, Student's *t*-test; Figures 6d–f). Taken together, these data demonstrated pivotal roles of RBP-J in the regulation of DA release in the striatum.

## Discussion

Here we provide evidence that Notch/RBP-J signaling regulates DA responsiveness in the striatum. Neuron-specific *RBP-J* deletion caused deficits in the active avoidance learning and enhanced responsiveness to apomorphine and SKF38393, a D1 agonist. The phenotypes of striatum-specific *RBP-J*-deficient mice are a mirror of what was observed in neuron-specific deletion of RBP-J. DA release in the striatum decreased in the absence of RBP-J. Abnormalities in active avoidance learning were restored by SCH23390, a D1 antagonist. These findings suggest the essential roles of Notch/RBP-J signaling in the regulation of DA signaling in the striatum.

Acquisition of the active avoidance response requires two types of learning: association learning between CS and an aversive US, and instrumental learning to prevent an US by behaviors performed during the CS presentation. DA is indispensable for both acquisition and maintenance of this conditioned avoidance.^37, 38, 40^ DA in the striatum is necessary for the acquisition of conditioned avoidance, whereas DA in the amygdala is indispensable only for acquisition of the avoidance.^46^ Neuron-specific loss of RBP-J affected both the acquisition and maintenance of conditioned avoidance, suggesting deficits in the striatum. This hypothesis was also confirmed by the results of the striatum-specific *RBP-J*-deficient mice. Furthermore, passive avoidance learning was intact in neuron-specific loss of *RBP-J*, suggesting normal functioning of the amygdala in the absence of RBP-J. Taken together, RBP-J in the striatum is indispensable for conditioned avoidance.

Response to the D1 agonist was enhanced in the absence of RBP-J. In addition, a low dose of SCH23390, a D1 antagonist, suppressed the conditioned avoidance impairment of neuron-specific RBP-J-deficient mice, although a D2 antagonist showed reduced effects on conditioned avoidance. In contrast, D1/D2 DA receptor expression was not changed in the absence of RBP-J. The number of dopaminergic neurons in the VTA and the SN decreased. Nonetheless, the effects may be negligible, because basal DA levels in the striatum were unchanged due to RBP-J deficiency. Acute DAPT treatment also impaired conditioned avoidance, suggesting that the effects of RBP-J deficiency on the responsiveness to a D1 dopaminergic agonist are direct, but not secondary to dopaminergic neuronal loss or developmental deficiencies. Furthermore, microdialysis experiments showed robustly reduced DA release in the RBP-J-deficient striatum, which was also confirmed by the reduced ratio of DOPAC to DA in the striatum. It remains to be elucidated how the Notch/RBP-J pathway underlies hyper-responsiveness to the D1 agonist in the striatum and associated behavioral changes. Notch/RBP-J pathway may affect DA release and/or intracellular dopaminergic receptor signaling in a cell autonomous mechanism.

Perturbation of Notch signaling may be associated with neuropsychiatric conditions. For example, loss of Aph1b/c, a component of γ-secretase, which is indispensable for Notch signaling, showed hypersensitivity to amphetamine and MK801, and enhanced DA turnover in the striatum, as do schizophrenic patients; this can be reversed by anti-psychotic treatment.^28^ Furthermore, genetic studies on *Drosophila* have indicated that a Notch signaling modulator, scabrous, regulates ethanol reward learning.^54^ In addition to multiple lines of biological support on the implication of Notch in higher brain functions, psychiatric genetics have provided promising, but still slightly conflicting, evidence that suggests a set of genes in Notch signaling to be involved in psychiatric conditions, such as schizophrenia (also see Supplementary Figures 3a–d).^3, 4, 5, 55, 56, 57, 58, 59, 60, 61, 62, 63, 64^ Together, the present study on Notch/RBP-J may provide novel insight not only in basic molecular signaling in the striatum but also in neurobiology of psychiatric conditions.
