# Supplementary material for: Regulation of striatal dopamine responsiveness by Notch/RBP-J signaling
Source: Transl Psychiatry. 2017 Mar 7;7(3):e1049–. doi: 10.1038/tp.2017.21 (PMC5416667; doi:10.1038/tp.2017.21)
Supplement: Supplementary Table 1 [file tp201721x1.docx]

Supplementary Table1

Primers and probes used for quantitative RT-PCR

| **Gene** | **Primer F** | **Primer R** | **Probe** |
| --- | --- | --- | --- |
| *Dopamine receptorD1* | 5’-tctggtttacctgatccctca-3’ | 5’-gcctcctccctcttcaggt-3’ | #82 |
| *Dopamine receptor D2* | 5’-tgaacaggcggagaatgg-3’ | 5’-ctggtgcttgacagcatctc-3’ | #17 |
| *Dopamine receptor D5* | 5’-tcctggtgtgcttatgctttc-3’ | 5’-tcagctaagaatcgtttggtttc-3’ | #18 |
| *Shh* | 5’-ccaattacaaccccgacatc-3’ | 5’-gcatttaacttgtctttgcacct-3’ | #32 |
| *Ptch1* | 5’-tgacaaagccgactacatgc-3’ | 5’-gtactcgatgggctctgctg-3’ | #64 |
| *Smo* | 5’-gcaagctcgtgctctggt-3’ | 5’-gggcatgtagacagcacaca-3’ | #3 |
| *Gli1* | 5’-ctgactgtgcccgagagtg-3’ | 5’-cgctgctgcaagaggact-3’ | #84 |
| *Gli2* | 5’-gcagactgcaccaaggagta-3’ | 5’-cgtggatgtgttcattgttga-3’ | #68 |
| *Gli3* | 5’-caccaaaacagaacacattcca-3’ | 5’-ggggtctgtgtaacgcttg-3’ | #71 |
| *GDNF* | 5’-tccaactgggggtctacg-3’ | 5’-gacatcccataacttcatcttagagtc-3’ | #70 |
| *Ret1* | 5’-tggagtttaagcggaaggag-3’ | 5’-acatctgcatcgaacacctg-3’ | #49 |
| *Gfrα1* | 5’-ttcccacacacgttttacca-3’ | 5’-gcccgatacattggatttca-3’ | #98 |
