# Supplementary figures and images for: Regulation of striatal dopamine responsiveness by Notch/RBP-J signaling
Source: Transl Psychiatry. 2017 Mar 7;7(3):e1049–. doi: 10.1038/tp.2017.21 (PMC5416667; doi:10.1038/tp.2017.21)

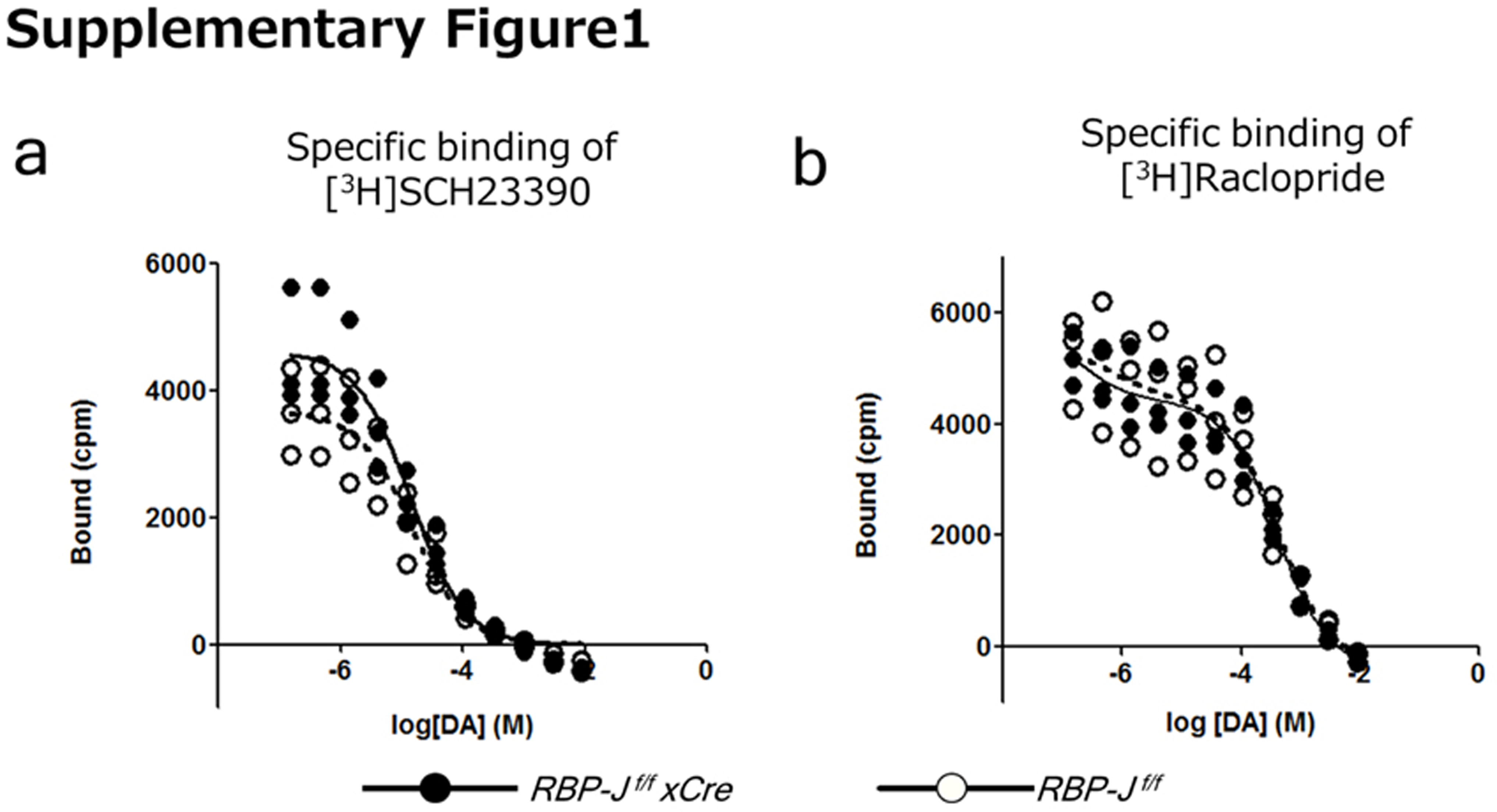

Supplement: Supplementary Figure 1 [file tp201721x2.tif]

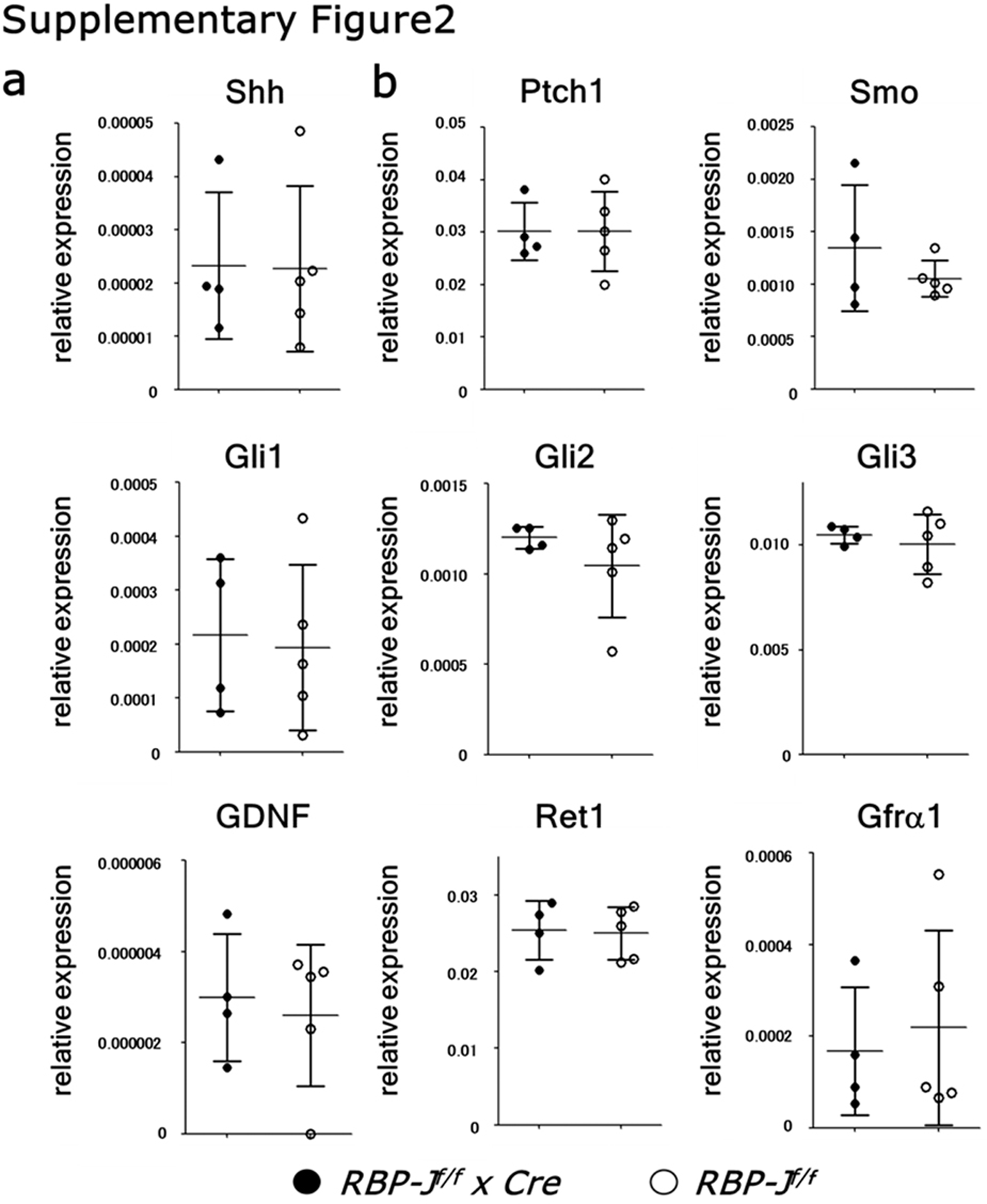

Supplement: Supplementary Figure 2 [file tp201721x3.tif]

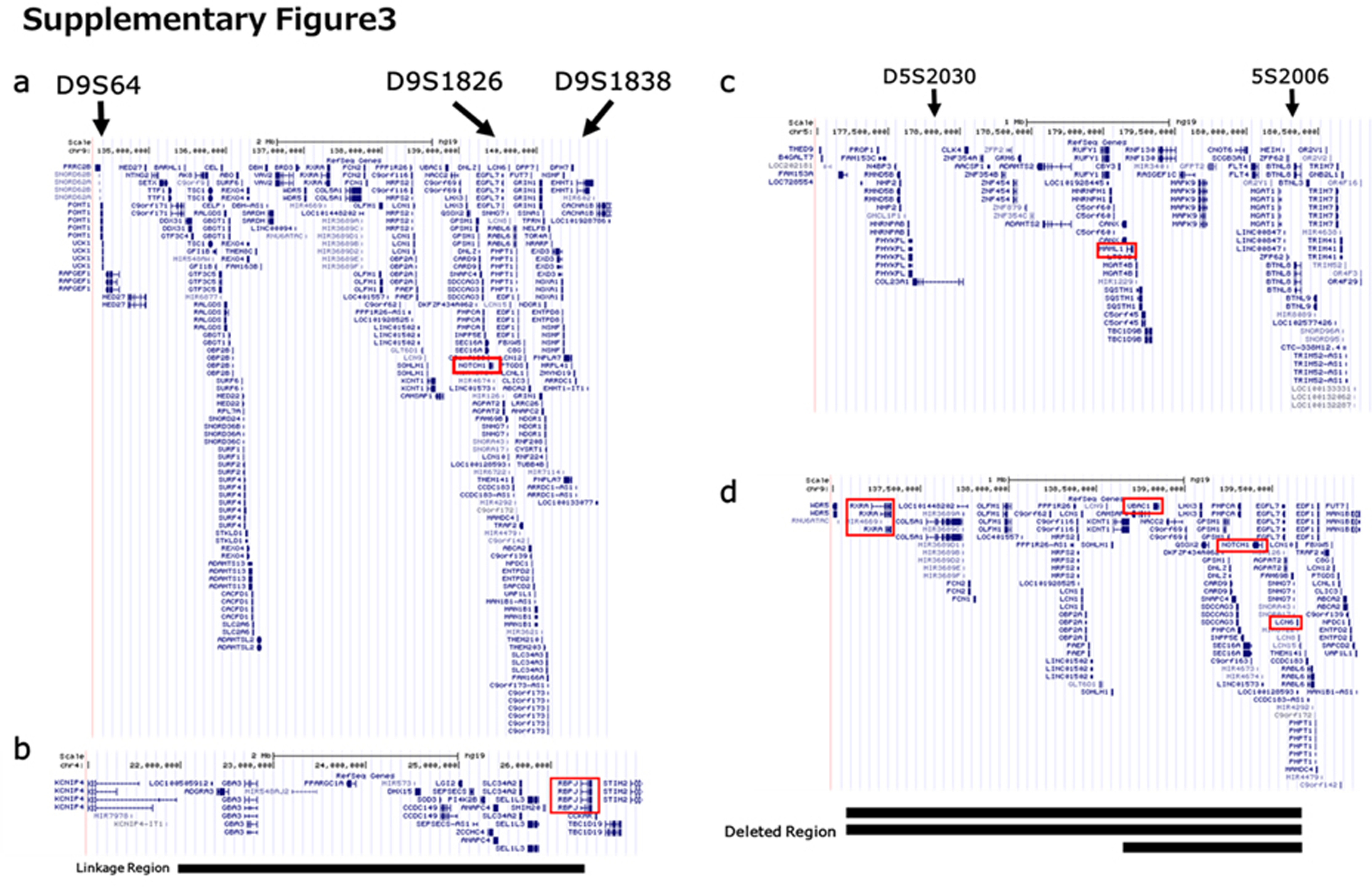

Supplement: Supplementary Figure 3 [file tp201721x4.tif]
