# Supplementary material for: Regulation of striatal dopamine responsiveness by Notch/RBP-J signaling
Source: Transl Psychiatry. 2017 Mar 7;7(3):e1049–. doi: 10.1038/tp.2017.21 (PMC5416667; doi:10.1038/tp.2017.21)
Supplement: Supplementary Figure Legends [file tp201721x5.docx]

**Supplementary Figure legend**

**Supplementary Fgure1**

Original dot plots of the dopamine competition assay of [^3^H] SCH23390 or [^3^H] raclopride binding (Figure 5e and f) are shown. Each point represents a single mouse.

**Supplementary Fgure2**

Shh-GDNF pathway was not affected in neuron-specific RBP-J-deficient mice.

Real-time PCR quantitation of *Shh, Ptch1, Smo, Glia, Gli2, Gli3, GDNF, Ret1 and Gfrα1* mRNA levels in the VTA (a) and the striatum (b) of *RBP-J ^f/f^ x Cre* or *RBP-J ^f/f^* mice. Results were normalized to GAPDH abundance.  Each point represents a single mouse, with the lines representing the mean ± s.d. of each group.

**Supplementary Fgure3**

Graphical representations of linkage regions including Notch-related genes.

The 9q34 (a, d), the 4p15 (b) and the 5q32 regions are shown. Black bars indicate a linkage region for schizophrenia, schizoaffective disorder and bipolar disease (chr4:21,993,262 to chr4:26,346,966) (b) and the deleted regions in three schizophrenic patients (d). Images were adapted from UCSC Genome Browser (Human GRCh37 Assembly (hg19) assembly) (<http://genome.ucsc.edu/>).

Genetic linkage studies and haplotype studies revealed that Notch/RBP-J signaling are linked with dopamine-related disease: from D9S64 to D9S1838 including NOTCH1 with substance abuse[^55^](file:///C:\Users\a.lobello\AppData\Local\Temp\Temp1_tp.2017.21.zip\2016TP000095\doc\2016TP000095-file001.doc#_ENREF_55) (a), from D9S1826 to D9S1838 near NOTCH1 with Parkinson’s disease[^56^](file:///C:\Users\a.lobello\AppData\Local\Temp\Temp1_tp.2017.21.zip\2016TP000095\doc\2016TP000095-file001.doc#_ENREF_56) (a), from chr4:21,993,262 to chr4:26,346,966 including RBP-J with schizophrenia, schizoaffective disorder or bipolar disease[^57^](file:///C:\Users\a.lobello\AppData\Local\Temp\Temp1_tp.2017.21.zip\2016TP000095\doc\2016TP000095-file001.doc#_ENREF_57) (b) and from D5S2030 to D5S2006 including MAML1 with schizophrenia[^58^](file:///C:\Users\a.lobello\AppData\Local\Temp\Temp1_tp.2017.21.zip\2016TP000095\doc\2016TP000095-file001.doc#_ENREF_58) (c). Rare chromosome micro-deletions in schizophrenic patients have been reported to contain NOTCH1[^59^](file:///C:\Users\a.lobello\AppData\Local\Temp\Temp1_tp.2017.21.zip\2016TP000095\doc\2016TP000095-file001.doc#_ENREF_59) (d). Genome-wide association studies suggests the association of Notch4 with schizophrenia[^3^](file:///C:\Users\a.lobello\AppData\Local\Temp\Temp1_tp.2017.21.zip\2016TP000095\doc\2016TP000095-file001.doc#_ENREF_3)^,^ [^4^](file:///C:\Users\a.lobello\AppData\Local\Temp\Temp1_tp.2017.21.zip\2016TP000095\doc\2016TP000095-file001.doc#_ENREF_4)^,^ [^60^](file:///C:\Users\a.lobello\AppData\Local\Temp\Temp1_tp.2017.21.zip\2016TP000095\doc\2016TP000095-file001.doc#_ENREF_60)^,^ [^61^](file:///C:\Users\a.lobello\AppData\Local\Temp\Temp1_tp.2017.21.zip\2016TP000095\doc\2016TP000095-file001.doc#_ENREF_61), although the results are still controversial[^5^](file:///C:\Users\a.lobello\AppData\Local\Temp\Temp1_tp.2017.21.zip\2016TP000095\doc\2016TP000095-file001.doc#_ENREF_5)^,^ [^62-64^](file:///C:\Users\a.lobello\AppData\Local\Temp\Temp1_tp.2017.21.zip\2016TP000095\doc\2016TP000095-file001.doc#_ENREF_62).
